# Understanding social risk factors of county-level disparities in COVID-19 tests per confirmed case in South Carolina using statewide electronic health records data

**DOI:** 10.1186/s12889-023-17055-y

**Published:** 2023-10-31

**Authors:** Fanghui Shi, Jiajia Zhang, Xueying Yang, Xiaowen Sun, Zhenlong Li, Sharon Weissman, Bankole Olatosi, Xiaoming Li

**Affiliations:** 1 South Carolina SmartState Center for Healthcare Quality, Columbia, SC USA; 2 Department of Health Promotion, Education, and Behavior, Arnold School of Public Health, University of South Carolina, Columbia, SC USA; 3 University of South Carolina Big Data Health Science Center, 915 Greene Street, Columbia, SC 29208 USA; 4 Department of Epidemiology and Biostatistics, Arnold School of Public Health, University of South Carolina, Columbia, SC USA; 5 Geoinformation and Big Data Research Lab, Department of Geography, College of Arts and Sciences, University of South Carolina, Columbia, SC USA; 6 School of Medicine, University of South Carolina, Columbia, SC USA; 7 Department of Health Services, Policy, and Management, Arnold School of Public Health, University of South Carolina, Columbia, SC USA

**Keywords:** Socioeconomic, County-level, COVID-19 testing, South Carolina

## Abstract

**Background:**

COVID-19 testing is essential for pandemic control, and insufficient testing in areas with high disease burdens could magnify the risk of poor health outcomes. However, few area-based studies on COVID-19 testing disparities have considered the disease burden (e.g., confirmed cases). The current study aims to investigate socioeconomic drivers of geospatial disparities in COVID-19 testing relative to disease burden across 46 counties in South Carolina (SC) in the early (from April 1, 2020, to June 30, 2020) and later (from July 1, 2020, to September 30, 2021) phases of the pandemic.

**Methods:**

Using SC statewide COVID-19 testing data, the COVID-19 testing coverage was measured by monthly COVID-19 tests per confirmed case (hereafter CTPC) in each county. We used modified Lorenz curves to describe the unequal geographic distribution of CTPC and generalized linear mixed-effects regression models to assess the association of county-level social risk factors with CTPC in two phases of the pandemic in SC.

**Results:**

As of September 30, 2021, a total of 641,201 out of 2,941,227 tests were positive in SC. The Lorenz curve showed that county-level disparities in CTPC were less apparent in the later phase of the pandemic. Counties with a larger percentage of Black had lower CTPC during the early phase (β = -0.94, 95%CI: -1.80, -0.08), while such associations reversed in the later phase (β = 0.28, 95%CI: 0.01, 0.55). The association of some other social risk factors diminished as the pandemic evolved, such as food insecurity (β: -1.19 and -0.42; *p*-value is < 0.05 for both).

**Conclusions:**

County-level disparities in CTPC and their predictors are dynamic across the pandemic. These results highlight the systematic inequalities in COVID-19 testing resources and accessibility, especially in the early stage of the pandemic. Counties with greater social vulnerability and those with fewer health care resources should be paid extra attention in the early and later phases, respectively. The current study provided empirical evidence for public health agencies to conduct more targeted community-based testing campaigns to enhance access to testing in future public health crises.

**Supplementary Information:**

The online version contains supplementary material available at 10.1186/s12889-023-17055-y.

## Background

Sufficient coverage of COVID-19 testing is an essential component of the COVID-19 public health response as it contributes to early case detection, self-isolation, and large-scale infection containment [1, 2]. In addition, adequate testing enables accurate recognition of disease burden in communities and contributes to appropriate responses at the public health system level (e.g., city shutdown) and individual level (e.g., face mask wearing and social distancing) [3]. Inequitable COVID-19 testing in marginalized populations may increase their risk of poorer health outcomes. Studies have revealed disparities in COVID-19 testing across geographic areas with different social and demographic characteristics. However, most of these studies rely solely on the COVID-19 testing rate relative to the whole population in an area and didn’t take the burden of disease (e.g., incidence, mortality, and hospitalization rate) into consideration [4–7]. An equitable allocation of COVID-19 testing should be defined as a relatively equal testing number in relation to disease burden instead of simply an equal number of tests per resident [8]. The World Health Organization (WHO) has suggested considering at least 10, and ideally 30, tests for every confirmed case in the early stage of the pandemic [9].

Examining socioeconomic risk factors of COVID-19 testing rate relative to disease burden beyond the individual level is needed for the implication of resource allocation and policy making. Investigation of the association of social factors with the aggregated health outcome disparities in an area enables us to know more about the underlying economic, environmental, and physical risk factors that affect the population’s vulnerability to disasters, including disease pandemics [10]. The Social Vulnerability Index (SVI), constructed by the Centers for Disease Control and Prevention (CDC), is an empirical place-based measurement of social vulnerability and contains four sub-indices representing four dimensions of social vulnerability, including socioeconomic status, household composition, racial/ethical minority, and housing type/transportation [4, 11]. Using SVI to explore social risk factors of low COVID-19 testing rates across counties seems prudent due to its multidimensional nature [4].

Apart from SVI, there are also some other social risk factors worth investigating, such as population health (e.g., obesity, diabetes, and life expectancy) and healthcare resources (e.g., the number of healthcare physicians per 100,000 population, insurance coverage, and the number of mental health providers) [4, 12]. These county-level socioeconomic factors may affect the COVID-19 testing rate relative to the disease burden, especially in more rural areas with potentially unique and complex socioeconomic challenges, such as South Carolina (SC) [13]. Racial composition of Black residents is another essential social determinant of health, and it can indicate the degree of racial/ethical residential segregation in an area [14]. Existing evidence has revealed that non-Hispanic Black communities are disproportionately affected by COVID-19 and tend to report higher incidence and mortality [15]. However, the literature regarding racial/ethnic disparities in COVID-19 testing has been limited and mixed [5, 16–18]. Some studies found higher testing/100,000 population in counties with more Black residents, while others found more tests in areas with more non-Hispanic White racial composition [5, 16, 17].

The association of social risk factors with COVID-19 testing relative to disease burden may vary across time during the pandemic in SC [5, 16, 17]. Limited laboratory COVID-19 testing was available at the beginning (shortly after February 28, 2020), and only individuals with symptoms and known exposures were eligible for testing [19]. Testing was not recommended for all close contacts of persons with COVID-19 infection until June 19, 2020 [20]. Thus, individuals were predominantly symptomatic testers in the pandemic's early phase (before July 2020). Additionally, with greater awareness of community inequalities in COVID-19 health outcomes as the pandemic evolves, more efforts addressing these inequalities emerged on the national, state, and local health agency levels [21]. All of these factors may lead to the variation of potential drivers of COVID-19 testing disparities in the early and later phases of the pandemic. However, there is a lack of empirical evidence regarding how disparities in COVID-19 testing changed as the pandemic evolved [18].

Leveraging statewide COVID-19 testing Electronic Health Record (EHR) data, the current study aims to explore the geographic disparities in COVID-19 testing in relation to disease burden in two phases of the pandemic (early phase: April 1, 2020, to June 30, 2020; later phase: July 1, 2020, to September 30, 2020) and to determine potential county-level social risk factors of these disparities in SC.

## Methods

### Data sources and linkage

All tests conducted by adult people (≥ 18 years old) that occurred between April 1, 2020, and September 30, 2021, in SC were included in this study. Their de-identified testing records about the testing date, diagnosis result, and race/ethnicity were extracted from statewide EHR data provided by the SC Department of Health and Environment Control (DHEC). If there were multiple testing records within one day for the same person, only one record was kept for those with the same testing results, and the positive one was kept for those with different testing results.

County-level social and demographic factors were derived from multiple publicly available datasets, such as the 2015–2019 American Community Survey (ACS) and the CDC. We linked the EHR data and county-level factors through each county's unique FIPS code. All the testing records with missing values in the FIPS code (about 2.7%) were excluded because they cannot be linked with the county-level data. The Institutional Review Boards at the University of South Carolina and relevant SC state agencies approved the proposal of this study.

### Time phases

The COVID-19 pandemic was divided into two phases based on the availability of COVID-19 testing resources for data analysis in the current study. The early phase (from April 1, 2020, to June 30, 2020) was when testing resources were limited to only high-risk groups (e.g., hospitalized patients with symptoms, healthcare facility workers, and patients over 65 years old) [17, 21]. Since July 2020, some no-cost events opened to the public, and anyone can get tested [22]. Thus, the second phase (from July 1, 2020, to September 30, 2021) was when the COVID-19 testing was available to the general population.

### Outcome

The primary outcome of this study is the monthly county-level COVID-19 tests per confirmed case (CTPC), and we defined a relatively equal number of CTPC across populations or areas as an indicator of equitable testing. CTPC was calculated as each county's monthly total COVID-19 testing divided by the monthly number of positive cases among the overall population, non-Hispanic Black and non-Hispanic White persons, respectively. The COVID-19 positive cases in the current study were defined based on the SC statewide Human Infection with 2019 Novel Coronavirus case report form (CRF) and included both lab-confirmed and probable COVID-19 cases. For the purpose of the data analysis in the current study, we also calculate the ratio of Black to White CTPC as Black CTPC being divided by White CTPC, with values larger than one indicating higher CTPC among the Black population.

Comparatively, a low CTPC means either a smaller number of COVID-19 tests or a high COVID-19 prevalence in the area. During a given time window, a relatively high value of CTPC in a specific geographic region could indicate a better chance of capturing asymptomatically or mild-symptomatically positive cases, which is a significant driver of the pandemic [2]. We acknowledge that the interpretation of CTPC is affected by the magnitude of positive cases, which serves as the denominator for the CTPC calculation. The CTPC would vary in magnitude with the variation of testing numbers when there are only a few new confirmed cases. However, the CTPC would be consistently low for an area with widespread infection, even with widespread testing. Thus, all analyses in the current study were based on the premise that the values of CTPC can be compared across locations or populations in a given time window but are not comparable between the early and the later phases of the pandemic when the number of new COVID-19 confirmed cases varied considerably in these two phases [23].

### Predictors

The 2018 overall SVI and four SVI sub-indices were directly extracted from the CDC’s Agency for Toxic Substances and Disease Registry website [24, 25]. To explore the relationship between SVI and CTPC disparities more granularly, 15 risk factors used to calculate the four sub-indices were extracted from the 2014–2018 ACS 5-year estimates dataset, including (1) Socioeconomic Status (poverty rate, unemployment rate, income per capita, and education level), (2) Household Composition and Disability (percentage of persons age 17 and younger, percentage of persons age 65 and older, single-parent household, and disability), (3) Racial/ethical Minority and Language (percent of people who are not non-Hispanic white, limited English speaking ability), and (4) Housing type and Transportation (percentage of structured housing with over nine units, mobile homes, housing units with more people than rooms, no vehicle, persons in group quarters) [24]. The overall SVI and four SVI sub-indices were generated by giving each county an overall rank and four theme ranks based on the 15 risk factors. They are all percentile ranks ranging from 0 to 1, with higher values indicating a greater social vulnerability.

Some other potential social risk factors of health disparities not included in the calculation of SVI were extracted from the 2015–2019 ACS 5-year estimates dataset and the Robert Wood Johnson Foundation’s 2020 county health rankings, including the Gini index of income inequality, percentage of persons under 65 years without health insurance, percent of workers aged over 15 years using public transportation to commute to work, food insecurity (percentage of the population who lack adequate access to food), percentage of people with diabetes, rate of obesity, number of primary care physicians, and the number of mental health providers. The detailed definitions and data sources of these variables were given in additional file 1.

Additionally, there are some common population-level confounders in the relationship between social risk factors and geographic disparities in COVID-19 health outcomes based on prior theoretical explanations, including (1) county-level population density (population estimate per square miles of land area), (2) urbanicity (the 2013 Rural–Urban Continuum codes extracted from the US Department of Agriculture), and (3) vaccination period (a dummy variable using April 1st, 2021, the day when COVID-19 vaccine was available to general population in SC, as a cutoff, with “0” indicating pre-vaccination period and “1” indicating after-vaccination period) [11].

### Statistical analysis

First, we used the 25^th^ percentile, median, 75^th^ percentile, and Interquartile Range (IQR) to describe the county-level characteristics of 46 counties in SC. Second, we generated two modified Lorenz curves to explore the county-level disparities in CTPC in the early and later phases of the pandemic [11]. The Lorenz curve was generated by plotting the cumulative proportion of positive cases on the x-axis and the cumulative proportion of tests on the y-axis in ascending order of CTPC at the county level, and the curves were color-coded by the percentage of non-Hispanic black residents in each county. The Lorenz curve would follow a straight 45-degree line with an equal distribution of CTPC across counties and become more convex with increasing inequity. The corresponding Gini and Hoover indexes (varying from 0 to 1) based on the Lorenz curves were computed to statistically indicate the extent of CTPC disparities across counties, with 0 representing perfect equality and 1 representing maximum inequality.

Third, we generated smoothed curves to further describe the temporal trend of average county-level CTPC among the overall, Black, and White populations. Last, we fitted generalized linear mixed effects regression models (GLMM) for the early and later phases to assess disparities in monthly CTPC. We examined the association of the overall SVI, four SVI sub-indices, and other county-level social risk factors with CTPC in separate models to prevent collinearity issues. Population density and urbanicity were included as confounders in all models, and the random intercept of county and time (in months) was included to account for repeated measures of each county over time. For the models conducted in the later phase, we add the vaccination period as an additional covariate to adjust the effect of vaccination availability to the general population. All analyses were performed in R statistical software version 4.1.2.

## Result

**Fig. 1:**
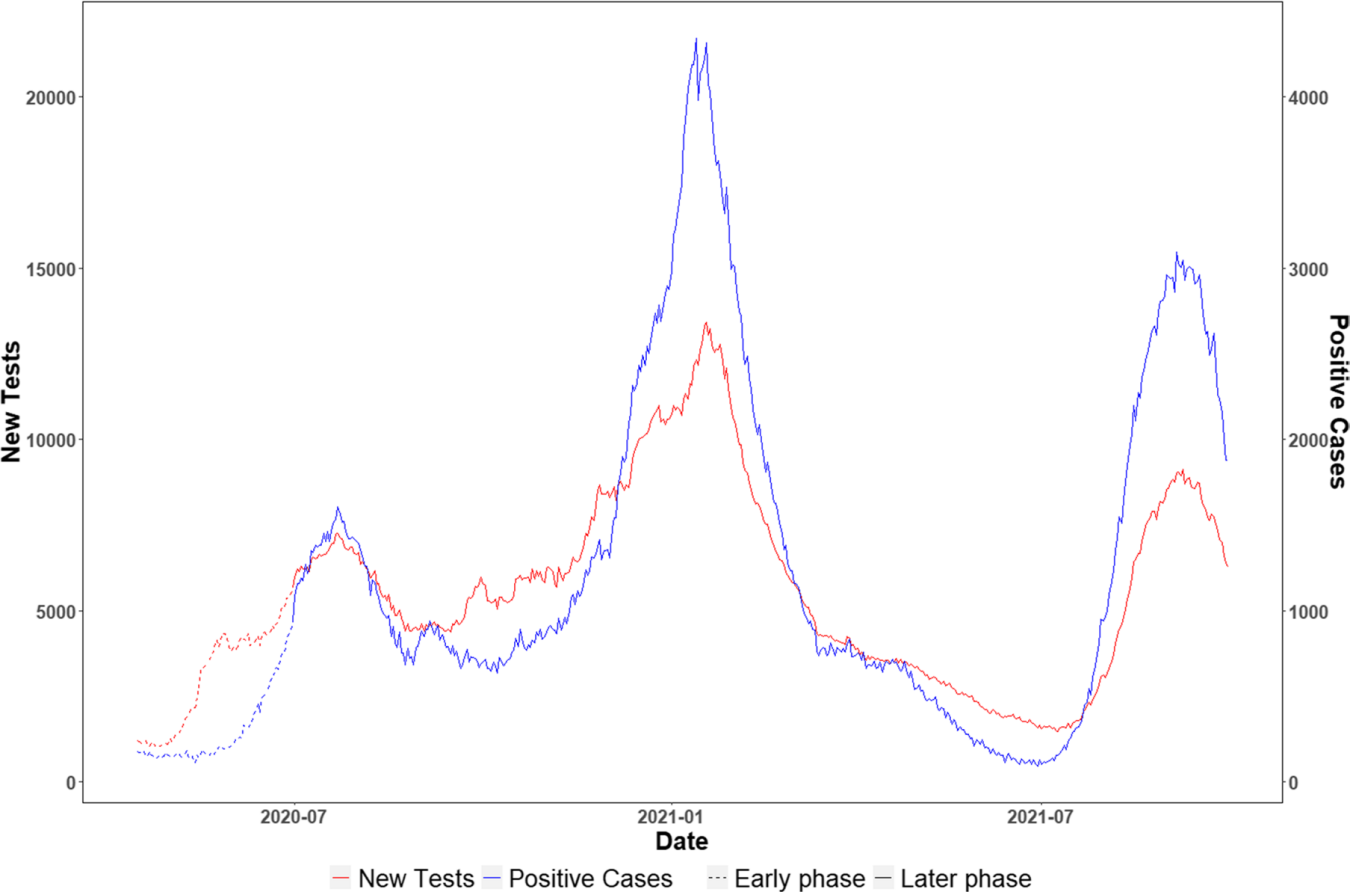
The number of new COVID-19 tests and positive cases among the overall population over time

From April 1, 2020, to September 30, 2021, a total of 2,941,227 tests were documented in SC, and 641,201 were positive. (Fig. 1) Table 1 describes the distribution of social and demographic characteristics across 46 counties in SC. In over half of the counties, more than 17% of individuals live below the US poverty level, and over 7% of the households have no vehicle access. The median proportion of Black was 31.64%, with the IQR being 23.3% (25^th^ percentile: 23.32, 75^th^ percentile: 46.61%). The median number of primary care physicians and mental health providers per 100,000 residents were 47.78 and 56.00, respectively.

**Table 1:** Descriptive statistics of county-level demographic and social factors across 46 counties in SC

Variable name	25th percentile	Median	75th percentile	IQR
**SVI sub-indices**				
*Theme 1: Socioeconomic status*				
Poverty (%)	13.97%	17.45%	19.94%	5.97%
Unemployed	5.51%	6.53%	7.95%	2.44%
Income	21325	24659	28396	7071
Less than high school (%)	12.83%	15.08%	19.06%	6.23%
*Theme 2: Household characteristics and disability*				
17 years or younger	19.80%	21.81%	23.19%	3.39%
65 years or older	16.28%	18.18%	20.01%	3.73%
Disability	8.24%	8.87%	10.52%	2.28%
Single parent household	32.21%	41.90%	47.38%	15.17%
*Theme 3: Minority status and language*				
Minority	31.77%	40.83%	54.73%	22.96%
Limited English proficiency	0.58%	0.97%	1.51%	0.94%
*Theme 4: Housing type and transportation*				
Multi-unit structure	1.21%	1.98%	4.45%	3.24%
Crowding	1.58%	2.07%	2.66%	1.08%
Mobile homes	17.82%	24.91%	31.29%	13.47%
Group quarters	1.41%	2.06%	3.57%	2.16%
No vehicle	5.84%	7.42%	9.06%	3.22%
**Other social risk factors**				
Black (%)	23.31%	31.64%	46.61%	23.30%
Income inequality	0.455	0.4735	0.4836	0.0286
No health insurance	9.41%	10.27%	11.04%	1.63%
Public transportation	24.00%	46.50%	78.00%	54.00%
Food insecurity	10.00%	11.50%	13.00%	3.00%
**Health care resources**				
Total primary care physicians	35.96	47.78	67.59	31.63
Mental health providers	23.25	56	261.5	238.25
**Underlying comorbidities**				
Diabetes	11.00%	13.00%	15.00%	4.00%
Obesity	36.00%	38.00%	41.00%	5.00%
Life expectancy	73.5	75.85	77.4	3.9

**Fig. 2:**
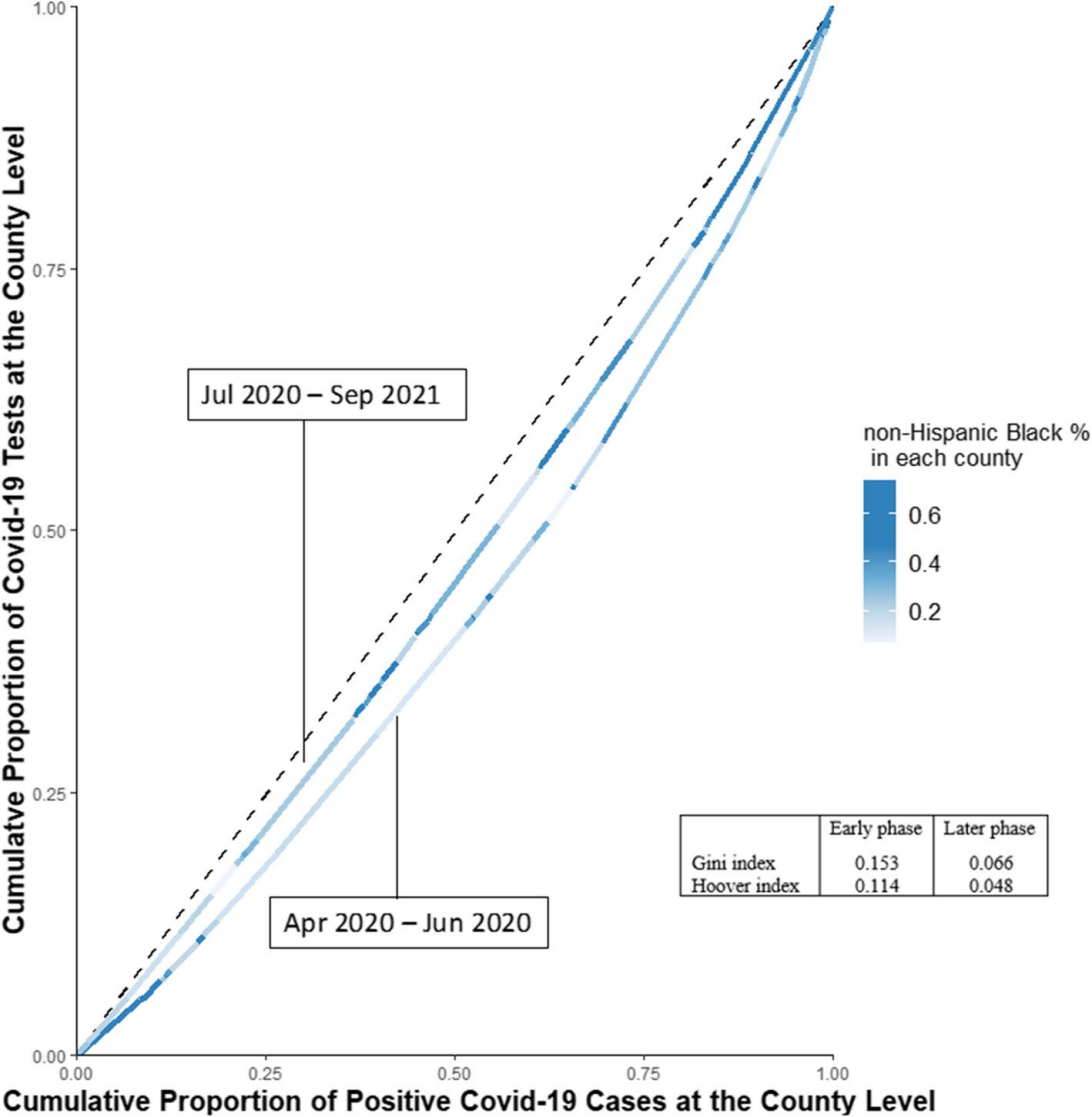
Lorenz curves of disparities in county-level COVID-19 testing relative to positive cases among the whole population in SC. Notes: The units of analysis are counties, and they are color-coded based on the proportion of Blacks in each county. Two separate curves were built for the early phase (April 1, 2020, to June 30, 2020) and the later phase (July 1, 2020, to September 30, 2021)

According to the two modified Lorenz curves depicting the distribution of COVID-19 CTPC across counties, a smaller magnitude of disparities was observed in the later COVID-19 phase than in the early phase. This was shown by the closer position of the second curve to the 45-degree line and the comparison of the Gini index (0.153 VS 0.066) and the Hoover index (0.114 VS 0.048) of the two curves. Additionally, counties with a higher proportion of Black residents (indicated by darker blue color) concentrated more on the lower half of the Lorenz curve for the early phase, suggesting that they were likely to report lower CTPC. Conversely, higher CTPC was more likely to be reported in counties with a higher proportion of Black residents in the later phase of the pandemic (Fig. 2).

**Fig. 3:**
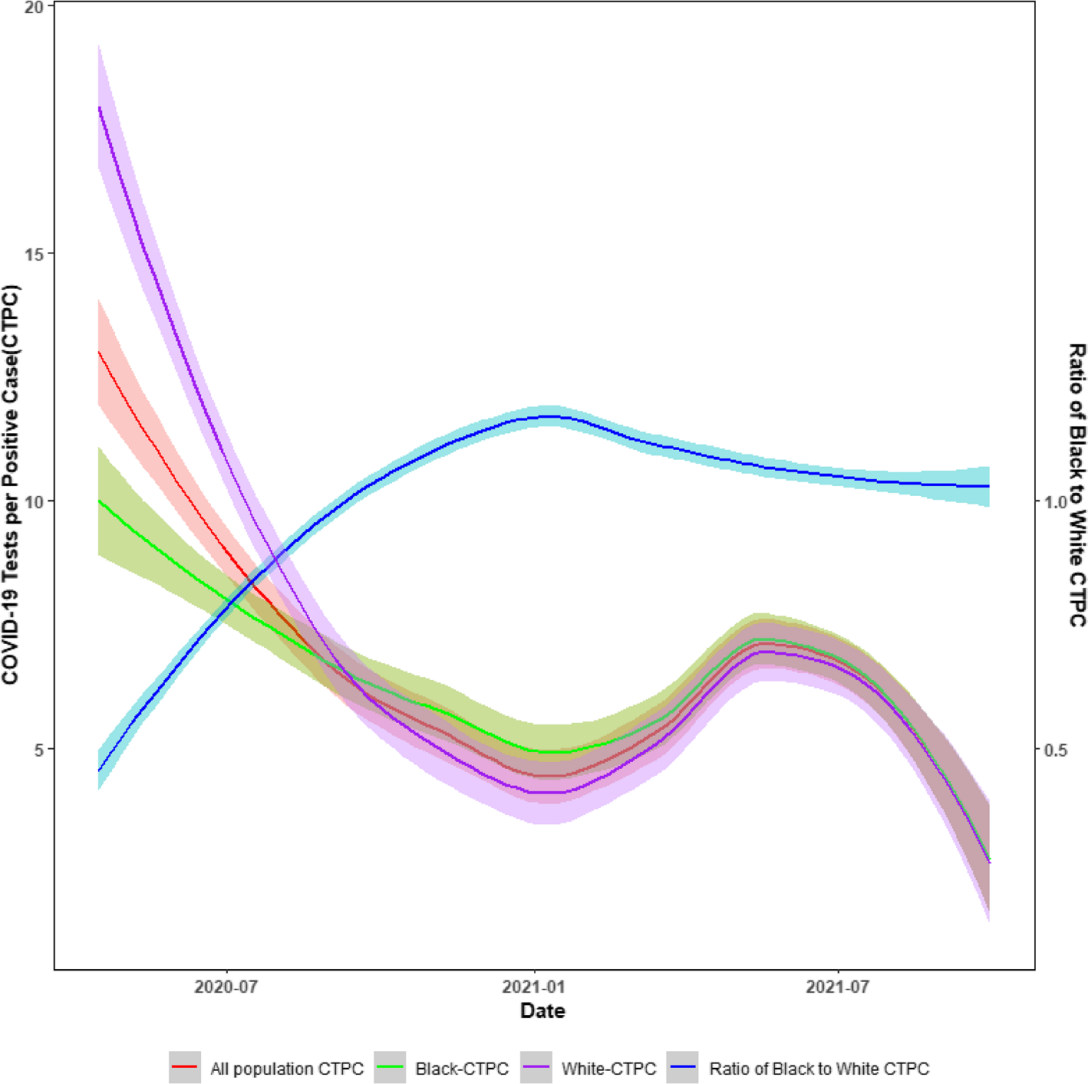
Monthly COVID-19 testing rate per positive case among overall, non-Hispanic Black and non-Hispanic White populations from April 1, 2020, to September 30, 2021, in SC

As shown in Fig. 3, compared to the White population, the CTPC among the Black remained lower until September 2020 and higher from September 2020 to May 2021. Eventually, the Black to White CTPC remained close to one at the end of the study period. Regarding the GLMM results, counties with greater vulnerability in the overall SVI (β = -1.55, 95%CI: -2.34, -0.76) and two SVI sub-indices, including the Socioeconomic Status (β = -1.57, 95%CI: -2.56, -0.57) and the Housing Type and Transportation (β = -0.85, 95%CI: -1.49, -0.21) had lower CTPC in the early phase of the pandemic. However, these associations became non-significant in the later phase (Table 2).

**Table 2:** Generalized linear mixed effects models^a^ of the overall SVI and the four SVI sub-indices associated with monthly COVID-19 tests per positive case in South Carolina

Factors	Early phase^b^		Later phase^c^	
	β (95% CI)	*p*-value	β (95% CI)	*p*-value
The overall SVI	-1.55(-2.34, -0.76)	< 0.001***	-0.07(-0.35, 0.21)	0.633
Theme 1: Socioeconomic status	-1.57(-2.56, -0.57)	0.002**	0.01(-0.33, 0.34)	0.975
Theme 2: Household characteristics and disability	-0.74(-1.49, 0.00)	0.051	-0.09(-0.34, 0.16)	0.478
Theme 3: Minority status and language	-0.51(-1.09, 0.08)	0.089	0.13(-0.06, 0.32)	0.186
Theme 4: Housing type and transportation	-0.85(-1.49, -0.21)	0.009**	-0.09(-0.31, 0.13)	0.419

*Abbreviations*: *SVI* social vulnerability index, *COVID-19* coronavirus disease 2019, *CI* confidence interval

^*^p < 0.05

^**^p < 0.01

^***^p < 0.001

^a^ Each index was put in a separate regression model to prevent collinearity issues; for all models, population density and urbanicity were controlled as confounders, and random intercepts of time (in month) and county were added controlling for repeat measures of each county; vaccination period was added as an additional confounder in models for the later phase

^b^ Early phase was from March 1, 2020, to June 30, 2020

^c^ Later phase was from July 1, 2020, to September 30, 2021

In the model examining the 15 individual SVI risk factors and some other social risk factors, we found varied relationships between these factors and CTPC across two phases (Table 3). First, there are some consistently significant risk factors across time. Counties with higher percentage of persons aged 17 and younger (β = -0.42, 95%CI: -0.72, -0.12) and higher percentage of food insecurity (β = -0.42, 95%CI: -0.71, -0.12) were more likely to have low CTPC. However, a high percentage of persons aged 65 years and older was associated with high CTPC (β = 0.55, 95%CI: 0.24, 0.86). Second, the significant relationship between some risk factors and CTPC disappeared across phases. For example, low CTPC was noted in counties with high diabetes rates (β = -1.60, 95%CI: -2.59, -0.60) only in the early phase. Third, we found that the percentage of Black and the percentage of minorities (all persons except non-Hispanic White) had reversed associations with CTPC in the early and later phases of the study period. In the early phase, the percentage of Black (β = -0.94, 95%CI: -1.80, -0.08) and other minorities (β = -0.99, 95%CI: -1.84, -0.15) were negatively associated with CTPC. However, these associations became positive in the later phase (percentage of Black: β = 0.28, 95%CI: 0.01, 0.55; percent of minorities: β = 0.28, 95%CI: 0.01, 0.54).

**Table 3:** Generalized linear mixed effects models^a^ of social risk factors associated with monthly COVID-19 tests per positive case in South Carolina

Factors	Early phase^b^		Later phase^c^	
	β(95% CI)	*P*-value	β (95% CΙ)	*P*-value
**SVI sub-indices**				
*Theme 1: Socioeconomic status*				
Poverty (%)	-2.07(-3.35, -0.78) **	0.002	-0.24(-0.63, 0.14)	0.217
Unemployed	-1.00(-2.23, 0.23)	0.112	0.13(-0.23, 0.49)	0.486
Income	2.02(1.11, 2.92)***	< 0.001	0.31(-0.06, 0.67)	0.103
Less than high school (%)	-1.35(-2.35, -0.35)**	0.008	-0.31(-0.63, 0.02)	0.066
*Theme 2: Household characteristics and disability*				
17 years or younger	-1.59(-2.34, -0.85)***	< 0.001	-0.42(-0.72, -0.12)**	0.005
65 years or older	1.51(0.74, 2.27)***	< 0.001	0.55(0.24, 0.86)***	< 0.001
Disability	0.74(-0.29, 1.77)	0.157	0.13(-0.23, 0.49)	0.477
Single parent household	-0.24(-1.15, 0.67)	0.608	0.21(-0.09, 0.50)	0.171
*Theme 3: Minority status and language*				
Minority	-0.99(-1.84, -0.15)*	0.021	0.28(0.01, 0.55)*	0.043
Limited English proficiency	-0.24(-1.39, 0.90)	0.676	0.11(-0.22, 0.45)	0.506
*Theme 4: Housing type and transportation*				
Multi-unit structure	0.65(-0.58, 1.88)	0.297	0.16(-0.26, 0.59)	0.446
Crowding	-1.47(-2.30,—0.64)**	0.001	-0.17(-0.42, 0.09)	0.213
Mobile homes	-1.93(-2.93, -0.94)***	< 0.001	-0.03(-0.39, 0.33)	0.868
Group quarters	0.43(-0.20, 1.06)	0.185	0.18(-0.04, 0.40)	0.111
No vehicle	-1.43(-2.60, -0.27)*	0.016	-0.13(-0.48, 0.23)	0.481
**Other social risk factors**				
Black (%)	-0.94 (-1.80, -0.08)*	0.0314	0.28(0.01, 0.55)*	0.0441
Income inequality	-0.28(-1.10, 0.55)	0.511	-0.01(-0.29, 0.28)	0.969
No health insurance	-1.43(-2.36, -0.49)**	0.003	-0.21(-0.52, 0.10)	0.184
Public transportation	-0.24(-1.17, 0.69)	0.612	0.15(-0.13, 0.44)	0.297
Food insecurity	-1.19(-2.17, -0.20)*	0.019	-0.42(-0.71, -0.12)**	0.005
**Health care resources**				
Total primary care physicians	0.63(-0.37, 1.62)	0.220	0.36(0.02, 0.69)*	0.039
Mental health providers	0.82(-0.62, 2.26)	0.263	0.50(-0.00, 1.01)	0.050
**Underlying comorbidities**				
Diabetes	-1.60(-2.59, -0.60)**	0.002	0.13(-0.20, 0.47)	0.429
Obesity	-0.70(-1.72, 0.33)	0.185	0.168(-0.18, 0.51)	0.343
Life expectancy	0.87(-0.13, 1.87)	0.088	0.11(-0.23, 0.44)	0.530

*Abbreviations*: *SVI* social vulnerability index, *COVID-19* coronavirus disease 2019, *CI* confidence interval

^*^p < 0.05

^**^p < 0.01

^***^p < 0.001

^a^ Each social risk factor was put in a separate regression model to prevent collinearity issues; for all models, population density and urbanicity were controlled as confounders, and random intercepts of time (in month) and county were added controlling for repeat measures of each county; vaccination period was added as an additional confounder in models for the later phase

^b^ Early phase was from March 1, 2020, to June 30, 2020

^c^ Later phase was from July 1, 2020, to September 30, 2021

## Discussion

Our analysis revealed the dynamic nature of county-level disparities in COVID-19 CTPC across time during the COVID-19 pandemic in SC. There are some consistent social risk factors (e.g., food insecurity and a higher percentage of persons aged 17 and younger) for low CTPC across the pandemic. However, some county-level socioeconomic factors (e.g., SVI, poverty, mobile homes, percentage of Black residents) significantly predicted CTPC at the early stage of the pandemic, but the associations disappeared (e.g., SVI, poverty, and mobile homes) or even reversed (e.g., the percentage of Black residents) as the pandemic evolved. Generally, these results highlight the systematic inequalities in COVID-19 testing, which were critical for controlling the pandemic. Additionally, the current study could provide empirical evidence and reference for public health officials to conduct more targeted community-based county-level COVID-19 testing campaigns.

In the early phase of the pandemic, counties with a larger percentage of Black residents had lower CTPC, which either indicated inadequate testing capacity in areas with a high density of Black populations or suggested a larger proportion of positive cases in these areas [2]. This finding mirrors previous studies using individual and aggregated-level data [2, 26–28]. In one study conducted in two Missouri regions, Black populations had consistently lower COVID-19 CTPC than White populations in the initial six months of the pandemic [2]. Black communities were disproportionally affected by the burden of COVID-19 disease. For optimal public health control, the number of COVID-19 tests should be scaled up correspondingly relative to the increased disease burden [29].

In the later phase of the pandemic, counties with a larger Black population had higher CTPC. One explanation was that Black residents had been over-represented in some frontline work in the healthcare sector and critical essential works (e.g., delivery workers, bus drivers, and restaurant workers) due to occupational segregation in the United States [29–31]. Regular screening testing was recommended by the CDC for racial/ethnic minority workers in high-density worksites or worksites with greater risk of exposure to co-workers or customers when the COVID-19 testing resources were more available [32]. Another potential explanation was that greater fear of infection and death due to being disproportionally influenced by COVID-19 made the Black population more likely to seek testing services [3, 33].

With the increasing availability of COVID-19 testing during the later phase, county-level disparities in CTPC decreased, and the association of other social risk factors with low CTPC diminished. Growing awareness of community disparities in COVID-19 testing and emerging national and regional efforts to mitigate these disparities may also play a critical role during this process [34–36]. At the beginning of the pandemic, testing resources were more available in counties with less social vulnerability, especially those showing less vulnerability in socioeconomic status, housing type, and transportation [4, 6, 17]. In late July 2020, expanding programs and practices for testing was set as one of the CDC’s priority strategies to reduce COVID-19 disparities and achieve health equity [37]. One specific strategy was making the information about COVID-19 available in multiple languages [37, 38]. In addition, community health workers, who serve as trusted local health care engagers, were involved in bridging medical distrust among racial/ethnical minorities [39, 40]. Thus, community health workers were instrumental in helping racial and ethnic minority populations navigate healthcare resources [41].

The various social risk factors of low CTPC at different pandemic stages found in our study can help public health and clinical officials identify to whom, where, and how resources should be targeted to contribute to a more effective pandemic response. In the early phase of the pandemic, counties with greater social vulnerability in terms of socioeconomic status (e.g., a large percentage of poverty and low income) and housing type and transportation (e.g., a large percentage of mobile homes, occupied housing units with more people than rooms, and households with no vehicle access) reported lower CTPC. However, counties with fewer primary care physicians and mental health providers per 100,000 residents were more likely to have low CTPC in the later phase. Targeted allocation of testing resources to counties with the greater SVI is crucial for suppressing onward disease transmission, especially in the early phase of the pandemic when the testing resources were limited [28, 30, 42, 43].

Some limitations of this study need to be acknowledged. First, we could not evaluate the ongoing dynamics of testing disparities as the testing data in SC was unavailable after October 2021, when the study was conducted. Second, our dataset cannot capture COVID-19 self-testing, which was popular among mild or asymptomatic persons in the later phase of the pandemic. Further testing at healthcare facilities was more likely to be conducted among those with positive home test results than those with negative results, which indicates the CTPC found in the later phase of our study may be underestimated. Information about the usage of COVID-19 self-testing kits should be collected in future studies to better understand the testing disparities in the later phase of the pandemic. Third, we focused our area-based study at the county level due to data availability, but each county's variations of the community-level factors should not be ignored. Last, equitable COVID-19 testing is defined in relation to the burden of confirmed cases in each county, and we acknowledge that this metric is affected by the disease burden. Still, our metric is in line with WHO’s guidance, and we believe our analysis can provide essential information for optimized testing strategies during the pandemic.

## Conclusions

Sufficient coverage of COVID-19 testing is critical for effective pandemic response, and equal allocation of limited testing resources in accordance with the disease burden is necessary for diminishing community disparities in health outcomes in the United States. We observed significant county-level disparities in CTPC and the variations of these disparities at different phases of the pandemic, underscoring the fact that for emergent crises, such as the COVID-19 pandemic, disparities are often dynamic. To improve the overall COVID-19 response, further efforts should focus on proactive strategies to address equity gaps in COVID-19 testing, and the focus of intervention efforts should be interpreted in the context of time and geolocations. Consistent inequalities in COVID-19 testing across time indicate that extra attention was needed for counties with greater social vulnerability, particularly for those with low socioeconomic status and housing or transportation barriers at the beginning of the pandemic. In contrast, more attention was needed for counties having fewer health care resources (e.g., primary care physicians and mental health providers) in the later phase.

### Supplementary Information

**Additional file 1.** The detailed description and data source of each variable.

## Data Availability

The datasets generated and/or analyzed during the current study are not publicly available due to provisions in our data use agreements with state agencies/data providers, institutional policy, and ethical requirements. We make access to such data available via approved data access requests.

## Abbreviations

SC: South Carolina
FIPS: Federal Information Processing Standards
CTPC: COVID-19 tests per confirmed case
WHO: World Health Organization
SVI: Social Vulnerability Index
CDC: The Center for Disease Control and Prevention
HER: Electronic Health Record
DHEC: Department of Health and Environment Control
ACS: American Community Survey
IQR: Interquartile Range
GLMM: Generalized linear mixed effects regression model
