# Supplementary material for: Understanding social risk factors of county-level disparities in COVID-19 tests per confirmed case in South Carolina using statewide electronic health records data
Source: BMC Public Health. 2023 Oct 31;23:2135. doi: 10.1186/s12889-023-17055-y (PMC10617158; doi:10.1186/s12889-023-17055-y)
Supplement: Supplementary file 1 — Additional file 1. The detailed description and data source of each variable. [file 12889_2023_17055_MOESM1_ESM.docx]

| Additional file 1 The detailed description and data source of each variable | |
| --- | --- |
| **Variable name** | **Description** |
| Population density | Population estimate divided by county area in square miles |
| Urbanicity | Beale’s Rural-urban code |
| **SVI** | The overall Social Vulnerability Index |
| **Socioeconomic status** | SVI subindex theme 1 |
| Poverty | Percentage of persons below US poverty level |
| Unemployed | Percentage of unemployed |
| Income | Per capita income |
| Less than high school | Percentage of persons with no high school diploma (age 25+) estimate |
| **Household characteristics and disability** | SVI subindex theme 2 |
| 17 years or younger | Percentage of persons aged 17 and younger |
| 65 years or older | Percentage of persons aged 65 and older |
| Disability | Percentage of civilian noninstitutionalized population older than 5 years with a disability estimate |
| Single parent household | Percentage of single-parent households with children under 18 |
| **Minority status and language** | SVI subindex theme 3 |
| Minority | Percentage of all persons except for non-Hispanic White |
| Limited English proficiency | Percentage of persons (age 5+) who speak English "less than well" |
| **Housing type and transportation** | SVI subindex theme 4 |
| Multi-unit structure | Percentage of housing in structures with 10 or more units |
| Crowding | Percentage of occupied housing units with more people than rooms |
| Mobile homes | Percentage of mobile homes |
| Group quarters | Percentage of persons in institutionalized group quarters |
| No vehicle | Percentage of households with no vehicle available |
| **Other social risk factors** |  |
| Income inequality | Gini index of income inequality |
| No health insurance | Percentage of persons under age 65 without health insurance |
| Public transportation | Percentage of workers age 16 or older using public transport (excluding taxicab) to commute to work |
| Food insecurity | Percentage of population who lack adequate access to food |
| Black (%) | Percentage of non-Hispanic black |
| **Health care resources** |  |
| Total primary care physicians | The number of primary care physicians per 100,000 |
| Mental health providers | The number of mental health providers per 100,000 |
| **Underlying comorbidities** |  |
| Diabetes | Percentage of people with diabetes |
| Obesity | Percentage of the adult population (age 18 and older) that reports a body mass index (BMI) greater than or equal to 30 kg/m2 (age-adjusted) |
| Life expectancy | The average period that a person may expect to live |
